# Reviewing the influence of positive leadership on worker well-being: A comprehensive analysis

**DOI:** 10.1016/j.heliyon.2024.e24134

**Published:** 2024-01-06

**Authors:** Edem M. Azila-Gbettor, Ben Q. Honyenuga, Eli A. Atatsi, Christina Naa Ayertso Laryea, Abigail Nana Konadu Quarshie

**Affiliations:** Department of Management Sciences, Ho Technical University, Ghana

**Keywords:** *Employee*, *Leadership*, *Leadership styles*, *Positive leadership*, *Systematic review*, *Well-being*

## Abstract

The study provides a review of existing empirical studies on the impact of positive leadership on worker well-being. The aim it to integrate current knowledge and provide directions for future research. The study analyses the content of 118 articles retrieved mainly from Google Scholar and Scopus database published between 2004 and 2022. Findings revealed that most of the studies are atheoretical with the dominant theory applied being conservation of resource theory. Furthermore, most of the studies were cross-sectionally designed, used convenient sampling and self-reported questionnaire. A conceptual framework is presented which synthesizes findings from prior works and shows the various dimensions of positive leadership practices and worker well-being. Additionally, a comprehensive future research agenda for theoretical and empirical advancement is suggested. The study offers a comprehensive framework that synthesizes and integrates the existing literature on positive leadership and worker well-being. The findings offer researchers in leadership a common platform for discourse.

## 1 Introduction

The dynamism of the global business landscape enjoins organizations, through their leaders, to create positive job settings for their workforce and robust relational teams through quality approaches, if they are to remain relevant and competitive [1]. A demonstration of good leadership in organizations is meant to influence employee attitude and behaviour, as well as encourage them to discover a sense of the meaningfulness of their work. Studies have found the exhibition of good leadership behaviour to support several employee outcomes such as motivation [2], work engagement [3], identification, and extra-role performance [4].

A model of leadership that has received scholarly attention is positive leadership. Positive leadership is not a distinct leadership concept, but rather an umbrella construct encompassing several leadership behaviours, including transformational, authentic, servant, ethical, etc. leadership styles [[5], [6], [7]]. Cameron [5] conceptualized positive leadership as the application of a variety of positive practices that assist individuals and organizations in accomplishing goals, succeeding at work, feeling elevated vitality, and achieving levels of effectiveness. Such leaders exhibit emotional aptitude and hopefulness in managing employees, empower and help employees to communicate, be responsible, and excel in their work [1,8]. Positive leadership behaviours have been found to promote the advancement of organizations that concentrate on utilizing strength-based methods [9].

One of the most explored issues in organizational behaviour continues to be the nature and impact of leadership [10]. Debatably, Kelloway and Francis [11] contend that the focus of scholarship in this domain is informed by positive outcomes between leadership behaviours and organizational relevant behaviours such as employee motivation, attitude and performance. Evidence from literature shows that studies on positive leadership behaviours and worker well-being have receive considerable scholarly attention. The growth of studies has been narrowed down on organizational benefits from employee well-being, as it has the tendency to propel organizational output, success, reduce costs and attrition rates [12,13]. Despite the popularity of the topic, there has not been a comprehensive review of existing studies specifically on the nexus between positive leadership behaviours and workers’ well-being. A cursory examination of current literature point to existing reviews on leadership behaviours [[14], [15], [16], [17]]. However, these studies were skewed in favour of specific leadership behaviours. The goal of the present study is to consolidate the various research streams by providing a systematic review of the effects of positive leadership behaviours on employee well-being in an organizational setting, by principally addressing the following research questions.

1. *RQ1*. What is the descriptive overview of studies on positive leadership and employee well-being? This question explores the yearly, continental, and country productivity of scholarship and the research design and analytical tools adopted in positive leadership and worker wellbeing, with the aim of identifying trends and patterns over time and providing insights in the methodologies employed and the validity of the findings.
2. *RQ2.* How is worker well-being and positive leadership measured in the research context? This question focuses on identifying different perspectives from which worker well-being and positive leadership has been conceptualized, with the aim of gaining a comprehensive understanding of how worker well-being and positive leadership are perceived and valued in different contexts.
3. *RQ3.* What theories are used to explain positive leadership and employee well-being relationship? This question offers a holistic review of the theoretical frameworks that have been used to explain the relationship between positive leadership and worker wellbeing, with the aim of gaining insights into the underlying mechanisms and processes through which positive leadership practices contribute to improved worker wellbeing.
4. *RQ4.* What is the outcome of positive leadership and employee well-being relationship? This question delves into the influence of positive leadership dimensions and moderating and mediating variables on worker well-being, aiming to uncover trends associated with their outcomes.

## 2 Methods

### 2.1 Review approach

The method adopted for the review followed the three-stage guideline of systematic review in terms of (i) planning the review, (ii) conducting the review and (iii) reporting of findings, proposed by Tranfield et al. [18]. In stage 1, the objectives and the protocol of the review were defined. In stage 2, a comprehensive literature-search and relevant information were extracted from the identified articles. Finally, the findings obtained from the collected data are consolidated and presented in stage 3 [19]. The choice of the research strategy was informed by the objective of providing a comprehensive coverage of up-to-date literature [[20], [21], [22]].

### 2.2 Data bases and keywords

Scopus and Google Scholar databases were used to conduct searches for the reviewed articles. The Scopus database has been extensively used in bibliometric research due to the comprehensive nature of its bibliographic data in relation to its timespan [23]. The use of Scopus further guarantees the inclusion of all significant and relevant empirical publications [24]. The decision to use Google Scholar is justified by its ability to retrieve articles that are more relevant and of superior quality in comparison to other search engines [25]. The abstracts, keywords and titles of publications in Google Scholar and Scopus were searched between 5th and 9th September, 2022 using terms such as “positive leadership” or “transformational leadership” or “servant leadership” or “authentic leadership”, “ethical leadership” etc. AND “worker wellbeing”.

### 2.3 Inclusion and exclusion criteria

Peer-reviewed journal articles published up until 2022 and in the English language were included in the study. Journals are well recognized for their role in disseminating verifiable information and are seen as having a significant influence within their respective domains [26,27]. Moreover, they are highly regarded for their significant contributions in creating and advancing the field of study within their specific framework [28]. The review excludes graduate theses, working papers, conference papers, books and book chapters, research notes, editorials, letters, commentaries and erratum. The appropriateness of the inclusion and exclusion criteria used in this study was deemed suitable, as they provide a reliable representation of relevant academic literature [29].

### 2.4 Data screening

**Fig. 1:**
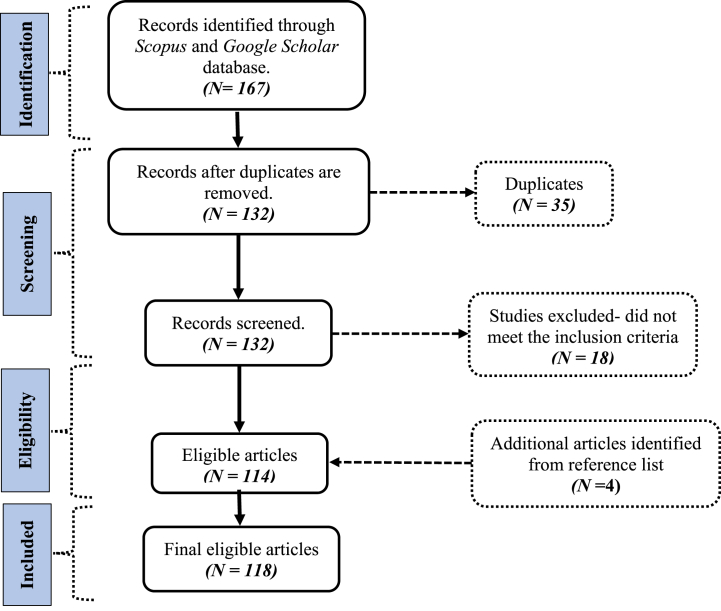
PRISMA flow chart for this study.

Source: *Adapted from Moher* et al. *(2009)*

The research returned 167 articles, 127 from Scopus and 40 from Google Scholar databases, respectively. After the removal of 35 duplicates, 132 studies were evaluated for legibility based on the abstracts. A total of 18 studies, made up of theoretical, review, graduate thesis, conference and working papers, editorials, letters, commentaries, and non-English articles, were further excluded from the study. Additional 4 studies were added, after cross-checking the reference list of eligible articles resulting in 118 studies. The information on eligibility assessment using Moher et al. [30] is presented in Fig. 1.

### 2.5 Analysis

A data extraction form was created in excel and used throughout the information retrieval process. The content of the form includes codes created to gather information on publication year and continent, research design, theory application, dimensions of positive leadership and worker wellbeing, reported outcomes of the relationship between positive leadership and worker wellbeing. The content from the coding categories was summarized and used to develop a conceptual framework shown in Fig. 5.

## 3 Results

### 3.1 Descriptive overview of studies

**Fig. 2:**
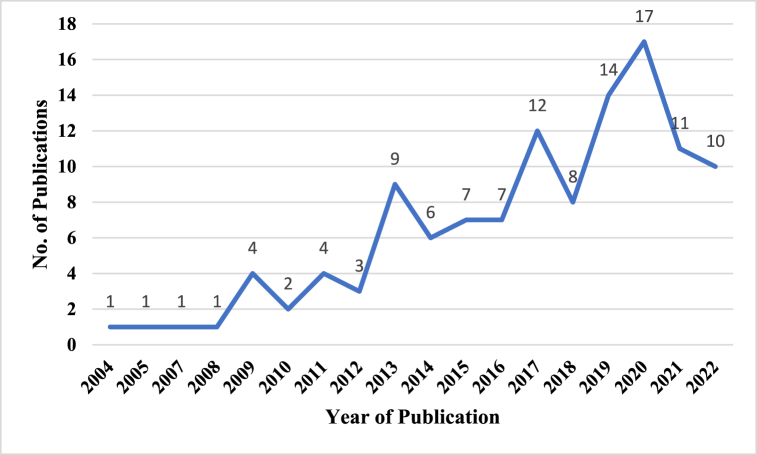
Yearly research production.

**Fig. 3:**
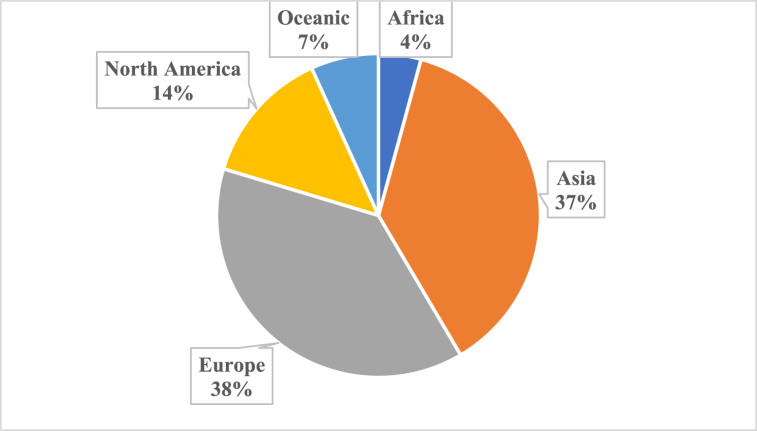
Continental production.

Yearly and continental productivity of scholarly work on positive leadership and well-being are reported in Fig. 2, Fig. 3*, respectively*. The findings suggest that the first study was published in 2004 and research output has since been erratic. Early years (from 2004 to 2016) witness low productivity of scholarly work. There was a marginal improvement after 2016, in the exception of 2018. The most prolific year of production is 2020 17 (14.4 %) followed by 2019 14 (11.9 %)(Fig. 2). Regarding continental production, Europe (38 %) and Asia (37 %) have the highest proportion of articles on positive leadership and employee wellbeing (see Fig. 3).

### 3.2 Research design

The descriptive analysis of research design is presented in Table 1. A quantitative approach was used in 94.1 % of the studies. Furthermore, 92.4 % of the studies adopted cross-sectional design, and 85.6 % of the studies were conducted at the individual level. Regarding sample techniques, 68.6 % used a non-probability sampling method. The convenience sampling (52.5 %) and simple random sampling (18.6 %) are the most common sampling techniques used. Similarly, a sample size of ≥300 (48.3 %) and 201–250 (23.7) were commonly used by the researchers. A greater proportion of the data was primary (99.1 %) in nature and collected using questionnaires (96.6 %) of various forms, such as online (25.4 %), self-reported (55.1 %) and structured (16.1 %) surveys.

**Table 1:** Research design.

Mode of Enquiry	Frequency	Percentage
Mixed method	3	2.5
Qualitative method	4	3.4
Quantitative method	111	94.1
Research Design		
Cross-Sectional	109	92.4
Longitudinal	9	7.6
Unit of Study		
Multi-level	5	4.2
Individual level	101	85.6
Organizational level	12	10.2
Sampling Technique		
Non-Probability	81	68.6
Probability	37	31.4
Sampling Type		
Convenience sampling	62	52.5
Purposive sampling	15	12.7
Simple sampling	22	18.6
Snowball sampling	4	3.5
Stratified sampling	15	12.7
Sample Size		
≤50	4	3.4
51–100	1	0.9
101–150	7	5.9
151–200	10	8.5
201–250	28	23.7
251–299	11	9.3
≥300	57	48.3
Data Collection Methods		
Interview and questionnaire	3	2.5
Observation	1	0.9
Online survey/questionnaire	30	25.4
Self-reported questionnaire	65	55.1
Structured questionnaire	19	16.1
Estimation Methods		
Content Analysis	4	3.4
Mean	64	54.2
Parametric test (*t-test, Anova, Manova,* etc.)	50	42.4
Correlation	67	56.8
Regression	26	22.0
CFA & EFA	42	35.6
PLS & CB SEM	51	43.2

Regarding analytical tools 54.2 % of the studies used mean and standard deviation and 3.4 % used content analysis. Multivariant analysis also received substantial attention. Exactly 43.2 % applied structural equation modeling; PLS SEM (37.3 %) and CB SEM (5.9 %), 22 % used regression analysis such as OLS regression (17.8 %), hierarchical (12.8 %). Given that the scales used were quite old and some were adapted, 27.1 % of the articles used CFA and EFA to validate the measures. Finally, 42.4 % used parametric inferential statistics such as chi-Square (8.5 %), anova 46 (14.4 %), Manova (10.1 %) and *t*-test (9.3 %).

### 3.3 Underpining theories

Within the realm of positive leadership and worker well-being research, researchers undertook an examination of research frameworks, guided by several theories. Of the 118 studies, 73 (62.0 %), used theories to explain the outcome of positive leadership and worker wellbeing. Additionally, the majority of the studies, which applied theories, used a single theory (71.2 %), followed closely by two theories (21.9 %).

**Fig. 4:**
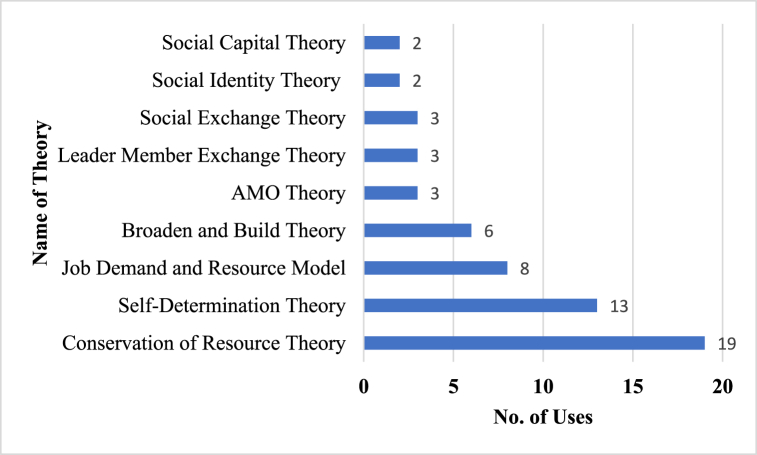
Type of theory.

The most dominant theories used are presented in Fig. 4. The conservation of resource theory [COR; 31], was used in 19 studies. The COR theory posits that individuals possess a finite pool of resources, and they aim to acquire, sustain, and safeguard these resources to reduce stress and maintain well-being [31]. The COR theory was used to conceptualize leadership as a resource in explaining positive wellbeing among employees [32,33]. Of course, good leadership provides followers with support, mentorship, guidance, opportunities for growth, advancement, and skill development, foster a positive and supportive work environment, enhance employees' self-efficacy, optimism, and motivation [34,35]. The availability of these opportunities functions as an invaluable resource that may assist people in managing stress and overcoming problems, therefore enhancing their overall well-being. Thirteen (13) studies used self-determination theory [SDT; 37]. The SDT posits that intrinsic motivation is nurtured by settings and social circumstances that promote autonomy, competence, and relatedness [36,37]. In the review for instance, Chen et al. [38] and Rahimnia and Sharifirad [39] adopted the SDT to explain how autonomy-supportive leadership, competence-enhancing leadership and relatedness-oriented leadership fulfills employees psychological need for internal motivation which directly affects their positive wellbeing. Eight (8) studies used job demand and resource model [JD-R; 41]. The JD-R is a theoretical framework used to comprehend the correlation between job attributes and the well-being of employees. The JD-R model states that excessive work expectations without enough resources may cause burnout, exhaustion, and health consequences. However, abundant job resources may mitigate job pressures and boost employee motivation, engagement, and well-being [40,41]. In the review, JD-R was used to contextualize leadership behaviours, including the provision of support empowerment, as a resource to explain the positive worker wellbeing outcome [42,43]. Six (6) studies used broaden and build theory [BBT; 46]. The BBT theory posits that positive emotions do not only make people happy; they shape their psychological, intellectual, and social resources over time. This resource buildup boosts individuals' resilience, social relationships, and well-being [44]. The BBT was used to explain how employees developed positive emotions through the leaders’ adoption of positive leadership behaviours and its effects on positive worker well-being [45,46]. Other less applied theories include ability-motivation-opportunity theories [47], social capital theory [48], social exchange theory [49], leader member exchange theory [50] and social identity theory [51].

**Fig. 5:**
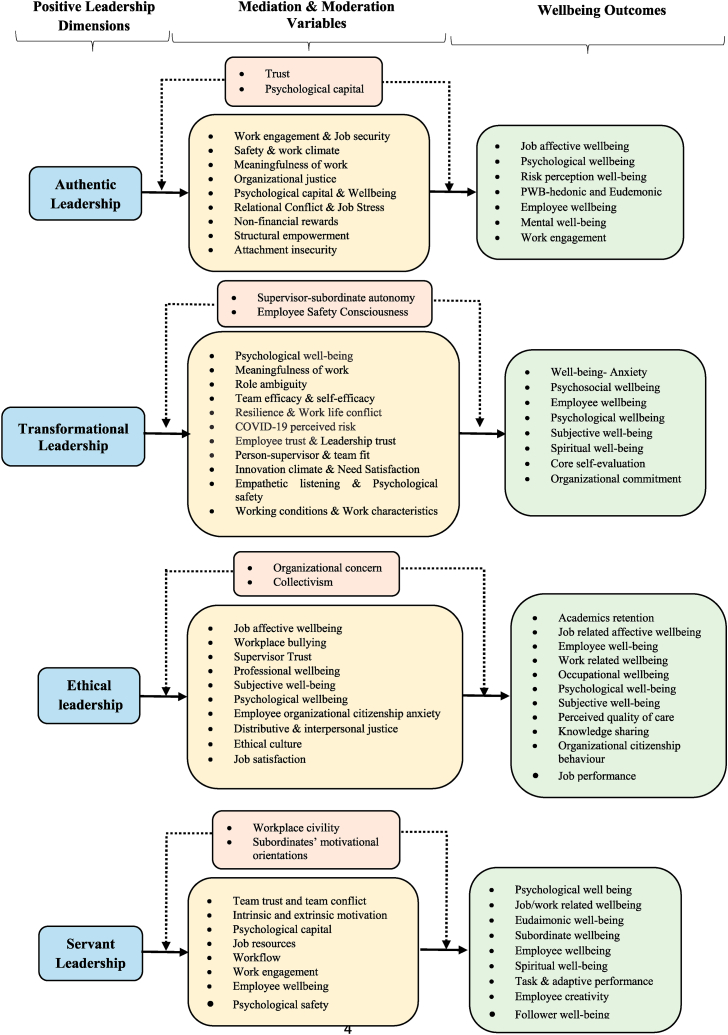
Impact of positive leadership on worker well-being.

### 3.4 Findings from positive leadership and worker well-being relationship

To provide a meaningful breakdown of the results reported by previous empirical studies, the researchers organized the positive leadership and worker well-being in a comprehensive table (Table 2) and framework (Fig. 5). Table 2 summarizes the dimensions of positive leadership (column 1), mediators (column 2), moderators (column 3), and outcomes of positive leadership (column 4). The thematic data structure, presented in Fig. 5, provides an integrative framework that captures diverse existing literature on the key variables. Findings from the framework reveal four leadership dimensions-authentic, transformational, servant, and ethical leadership, with several areas of worker well-being such as psychological, employee, mental, subjective, spiritual, occupational, subordinate, and spiritual, among others. Each leadership dimension is presented with an outcome, mediating and moderating variables. The information in the table is analyzed to provide trends that emerged from published articles.

**Table 2:** Summary of selected Positive Leadership and Worker Well-being Studies.

Dimensions of Positive Leadership	Mediators of Positive Leadership and Worker well-being	Moderators Positive Leadership and Worker well-being	Outcomes of Positive Leadership
Authentic Leadership Adil and Kamal [32]; Bannay and Hadi [52]; Birkeland Nielsen et al. [53]; Cassar and Buttigieg [54]; Farr-Wharton et al. [55]; Salleh et al. [56]; Kim et al. [57]; etc.,	• Work engagement -Adil and Kamal [32]; • Job security -Bannay and Hadi [52]; • Safety/work climate- Birkeland Nielson et al. [53]; • Meaningfulness of work-Cassar and Buttigieg [54]; • Organizational justice- [58]; • Relational conflict- [59]; • Job stress- [59]; • Non-financial rewards-Salleh et al. [56]; • Structural empowerment- [48]; • Attachment insecurity- [39]; • Wellbeing: life, workplace and psychological- [60];	• Psychological capital- Kim et al. [57];	• Job affective wellbeing-Adil and Kamal [32] • Psychological wellbeing-Bannay and Hadi [52]; Kim et al. [57]; • Risk perception well-being-Birkeland (Nielson et al. [53]; • PWB-hedonic and Eudemonic-Cassar and Buttigieg [54]; Farr-Wharton et al. [55]; • Mental well-being- [48]; • Work engagement- [60]; • Employee well-being- Salleh et al. [56];
Transformational Leadership Kelloway et al. [61]; Arnold and Wash [62]; Berger et al. [43]; Can [63]; Djourova et al. [64]; Geibel et al. [65]; Irshad et al. [66]; Jacobs et al. [67]; Mehdinezhad and Nouri [68]; Tafvelin et al. [69]; Munir et al. [70]; etc.,	• Psychological well-being-Ding et al. [71]; • Meaningfulness of work-Ding et al. [71]; • Role ambiguity-Berger et al. [43]; • Self-efficacy-Djourova et al. [64]; Liu et al. [72]; • Team and self-efficacy-Nielsen and Munir [73]; • Resilience-Djourova et al. [64]; • Covid-19 perceived risk-Irshad et al. [66]; • Employee trust-Irshad et al. [66]; • Leadership trust-Kelloway et al. [11]; Liu et al. [72]; • Fit- person-supervisor & team fit-Klaic et al. [74]; • Innovation climate-Tafvelin et al. [69]; • Need Satisfaction-Stenling and Tafvelin [75]; • Working conditions-Nielsen and Daniels [51]; • Work-life conflict-Munir et al. [70];	• Supervisor-subordinate autonomy-Berger et al. [43]; • Employee safety consciousness-Irshad et al. [66];	• Well-being- Anxiety-Berger et al. [43]; • Psychosocial wellbeing-Can [63]; • Employee wellbeing-quality of work life, quality of life-Kara et al. [76]; • Employee wellbeing -positive affective wellbeing-Kelloway et al. [10]; • Psychological wellbeing-Kelloway et al. [61]; Irshad et al. [66]; Munir et al. [70]; • Employee wellbeing-job satisfaction, and work-related strain-Klaic et al. [74]; • Negative affective well-being-perceived work stress-Liu et al. [72]; • Subjective well-being-Nielsen and Daniels – [51]; • Spiritual well-being-Mehdinezhad and Nouri [68]; • Core self-evaluation-Ding et al. [71]; • Well-being-psychosomatic complaints and psychological distress-Djourova et al. [64];
Ethical leadership Ahmad et al. [33]; Chughtai [49]; Gilet et al. [77]; Hayat Bhatti et al. [78]; Hendriks et al. [79]; Sarwar et al. [80]; etc.,	• Job affective wellbeing-Ahmad et al. [33]; • Workplace bullying-Ahmad et al. [33]; • Supervisor Trust-Chughtai et al. [49]; • Professional wellbeing-Gilet et al. [77]; • Subjective well-being- Hayat Bhatti et al. [78]; • Trust-Huang et al. [81]; • Psychological wellbeing-Huang et al. [81]; • Employee organizational citizenship anxiety-Fu et al. [82]; • Distributive & interpersonal justice-Li et al. [83]; • Ethical culture-Sarwar et al. [80]; • Job satisfaction-Yang [84];	• Organizational concern-Fu et al. [82]; • Collectivism-Li et al. [83];	• Academics retention-Ahmad et al. [33]; • Job related affective wellbeing-Ahmad et al. [33]; • Employee well-being-work engagement and emotional exhaustion-Chughtai et al. [49]; • Occupational wellbeing-Li et al. [83]; • Subjective well-being -Happy Workers & Life Happiness-Yang [84]; • Perceived quality of care (Gilet et al. [77]; • Knowledge sharing- Hayat Bhatti et al. [78]; • Organizational citizenship behaviour-Huang et al. [81];
Servant Leadership Chen et al. [38]; Clarence et al. [45]; Coetzer et al. [85]; der Kinderen et al. [86]; Donia et al. [87]; Jin et al. [88]; Obi et al. [89]; etc.,	• Team trust and team conflict (Obi et al., 2021; • Intrinsic and extrinsic motivation-Chen et al. [38]; • Psychological capital-Clarence et al. [45]; • Job resources-Coetzer et al. [85]; • Workflow-Jin et al. [88]; • Work engagement-Jin et al. [88]; • Employee wellbeing-Kaltiainen and Hakanen [46]; • Team trust- Obi et al. [89];	• Workplace civility-der Kinderen et al. [86]; • Subordinates' motivational orientations-Donia et al. [87];	• Psychological well-being-Clarence et al. [45]; • Job/work related wellbeing-Coetzer et al. [85]; Jin et al. [88]; • Eudaimonic well-being-Chen et al. [38]; der Kinderen et al. [86]; • Subordinate wellbeing-job satisfaction-Donia et al. [87]; • Employee wellbeing • Spiritual well-being -Obi et al. [89]; • Task & adaptive performance-Kaltiainen and Hakanen [46];
Others			
HRM Responsible leadership-He et al. [90];	• Employee wellbeing-He et al. [90];		• Task performance-He et al. [90];
Virtuous leadership-Hendriks et al. [79];	• Trust-Hendriks et al. [79]	• Leader virtues- Hendriks et al. [79]	• Work related wellbeing-job satisfaction- Hendriks et al. [79]
Humble leadership-Luu [91];	• Job crafting- Luu [91];		• Employee wellbeing -Luu [91];
Positive leadership--Adams et al. [50]; Respectful leadership Inclusive leadership-Kelloway et al.- [10];		• Discrimination and inclusion-Adams et al. [50];	• Work related well-being -Adams et al.- [50]; • Employee wellbeing-mental health, job satisfaction, positive affective wellbeing-Kelloway et al. [10];

### 3.5 Positive leadership dimensions

Roughly about 47 % of the studies reviewed focused on transformational leadership. Most of the studies examined transformational leadership as a predictor variable, with only 3 studies using it as a mediating variable [[62], [92], [93]] and 1 study as a moderating variable [47]. Authentic leadership was used in 28 % of the studies [32,52], ethical leadership in 12 % of the studies [49,94], and servant leadership in 8 % of the studies [38,45,85]. Other positive leadership styles used in well-being research include respective and inclusive leadership [50], humble leadership [91] and virtuous leadership [79]. The analysis of the landscape indicates a notable focus on transformational leadership in studies pertaining to various aspects of worker well-being. However, it also highlights a need for more comprehensive exploration in less-explored leadership, to gain a more nuanced understanding of their roles in fostering worker well-being.

### 3.6 Worker well-being dimensions

The concept of wellbeing is based on a number of psychological theories, including existential [95], social [96], humanistic [97], developmental [98], and clinical [99] psychology. Worker well-being refers to the holistic state of an individual in the workplace, encompassing various dimensions of health, satisfaction, and functioning [100,101]. The concept encompasses several dimensions, namely physical, mental, emotional, and social, which together influence an individual's holistic well-being in the context of their professional setting [102,103].

Wijngaards et al. [104] have espoused four key construct dimensions, including the philosophical foundation, scope, temporary stability and valence, along which worker well-being has been operationalized in the existing literature. The review indicates measurement of worker-well-being across these dimensions. The philosophical tradition of eudaimonia and hedonic [105] has been adopted for instance by Cassar and Buttigieg [54] and Farr-Wharton et al. [55] and covers the realization of human potential and subjective experience of happiness [55,106]. Temporal stability, that is a state-like and trait-like dimension, has received attention from other scholars including anxiety and stress [43,58], risk perception well-being [51]. It is indicative to know that the state-like constructs exhibit significant fluctuations over time, as a result of their elevated state variance, while trait-like constructs show better consistency and stability over time [107]. In terms of scope-dimensions, context-free and domain-specific constructs including job satisfaction, work-related strain, organizational commitment etc. have been measured [[72], [74], [108]]. The constructions that are context-free pertain to the overall lives and experiences of workers, whereas the constructs that are domain-specific focus on well-being within certain areas of life [109]. Finally, other scholars measure valence dimension including positive affective, employee wellbeing, work engagement [10,91,76,60]. In essence, this review demonstrates the complexity and richness of worker wellbeing assessment, emphasizing the need to consider diverse dimensions and constructs when evaluating and promoting wellbeing in the workplace.

### 3.7 Findings of the relation between positive leadership and worker well-being

Numerous reviewed studies assessed the impact of dimensions of positive leadership on worker well-being. In the exception of studies of Liu et al. [60], Perilla-Toro and Gómez-Ortiz [110], majority of the studies reported transformational leadership to positively impact all forms of worker well-being [[61], [66], [67]]. For instance, Kara et al. [76] found transformational leadership has a significant positive influence on hotel employees' perceived quality of work and life. Servant leadership was also found to positively predict all forms of worker well-being. For example, Obi et al. [111] reported servant leadership to positively foster spiritual well-being while Jin et al. [88] reported a significantly positive relationship between servant leadership and work-related well-being. Additionally, ethical leadership was found to significantly influence all forms of worker well-being, in the exception of the study of Yang [84]. For example, Teimouri et al. [112] and Li et al. [83] reported a significantly positive relationship between ethical leadership and employee psychological well-being and occupational well-being, respectively. Finally, in the exception of a few studies that confirm a negative relationship between authentic leadership and worker well-being [39,58], majority of articles reviewed including Weiss et al. [113] and Wang and Jin [59] found authentic leadership to significantly reduce employees’ level of stress and enhances employees psychological well-being, respectively. In general, the aforementioned results underscore the favourable impacts of direct positive leadership styles, including transformational, servant, and ethical leadership, on several aspects of employee well-being. Nevertheless, the nuances in a few of the studies indicate that the relationship might not always be universally positive, as seen in the case of authentic and transformational leadership where conflicting results exist. This highlights the need for further research to examine the conditions or contexts in which various positive leadership styles may have either a beneficial or negative influence on the well-being of workers.

### 3.8 Mediating and moderating variables in positive leadership and worker well-being relation

The articles reviewed also included moderating or mediating variables in examining the effect of positive leadership on worker well-being. Exactly 56 % and 14 % of the reviewed articles assessed the impact of mediating and moderating variables on positive leadership-worker well-being relation, respectively. The mediating effect was examined using worker well-being and other variables. Majority of mediating effects predicting worker well-being were found to be positive and significant. For example, Cassar and Buttigieg [54] found the meaningfulness of work to significantly mediate the relation between authentic leadership and hedonic and eudaimonic well-being. In another study, Berger et al. [43] found that the relationship between transformational leadership and anxiety was fully mediated by role ambiguity and team climate. Additionally, studies that used well-being found it to positively mediate the relation between positive leadership and outcome variables. For example, Koon and Ho [60] found well-being to positively mediate the relation between authentic leadership and work engagement. Furthermore, Jian et al. [108] found well-being partially mediates the relationship between transformational leadership and employees’ organizational commitment.

All moderating effects predicting worker well-being were found to be positive and significant [43,66,82]. For instance, Adil and Kamal [32] found psychological capital to positively moderate the relation between authentic leadership and job-related affective well-being. Finally, Arnold and Wash [62] found transformational leadership to moderate the relationship between customer incivility and employee well-being.

## 4 Conclusions

### 4.1 Discussions

In today's dynamic and demanding work environments, the general well-being of workers plays a crucial role in shaping productivity, job satisfaction, and the overall effectiveness of the organization [114]. In recent years, organizational psychology and management studies have focused on how positive leadership styles affect worker well-being. This study provides a comprehensive review of the influence of positive leadership on the well-being of workers across diverse industries and organizational settings. The review spans 2004 to 2022 and was based on 118 eligible empirical studies, after a thorough data-screening process based on Moher et al. [30] approach.

In relation to research question 1, the descriptive overview offers significant insights into the field of positive leadership and well-being research, including an overview of trends, geographical variations, methodological decisions, and prospective avenues for enhancing future investigations. The scholarly work has shown fluctuating productivity since its inception. Early years (2004–2016) were characterized by low output, with a marginal increase in post-2016, except for 2018. The year 2020 saw the highest level of productivity, constituting 14.4 % of the total productivity. The field's initial slow progress and subsequent erratic growth indicate a maturing research landscape. The increase seen in 2020 might perhaps be attributed to the expanding acknowledgement of the significance of positive leadership, particularly in periods within organizations characterized by difficulties and obstacles. Furthermore, the global pandemic in 2020 may have also played a role in driving organizations to prioritize productivity and seek effective leadership strategies to navigate through challenging times.

Europe and Asia are at the forefront in terms of scholastic production. These two continents have a rich history of academic excellence and have been home to some of the world's oldest and most prestigious universities. Additionally, Europe and Asia have consistently invested in research and development, fostering a culture of innovation and intellectual growth. The geographical focus on research in Europe and Asia further indicates a distinct regional interest, may be influenced by variations in organizational and cultural factors pertaining to leadership and well-being approaches.

A predominant quantitative approach (94.1 %) is observed, with cross-sectional designs (92.4 %) being popular. Studies mainly focus on individual-level analysis (85.6 %). Non-probability sampling (68.6 %) is common, often using convenient (52.5 %) methods. These findings suggest that the majority of research in this field relies heavily on numerical data and static observations. However, it is important to note that there is a need for more diverse research methods, such as longitudinal studies or qualitative approaches, to provide a comprehensive understanding of the topic. The reliance on convenience sampling may raise questions about generalizability.

Primary data collection is prevalent (99.1 %), mainly through questionnaires (96.6 %). The extensive use of primary data and questionnaires, especially through online platforms and self-reporting methods, indicate a dependence on participant perspectives and self-evaluation, potentially influencing the reliability of findings. Mean and standard deviation (54.2 %) are commonly used, along with advanced techniques like structural equation modeling (43.2 %), parametric inferential statistics (42.4 %) and regression analysis (22 %) are also prevalent. Confirmatory and exploratory factor analyses (27.1 %) are employed to validate measures. The diverse analytical tools employed, from basic statistics to advanced modeling, showcase the methodological richness in the field. The use of both confirmatory and exploratory factor analyses indicates a commitment to ensuring measurement validity.

Regarding research question 2, the findings suggest that researchers measured worker well-being through diverse lenses, including philosophical concepts like human potential and happiness, considering fluctuations over time, differentiating between broader life experiences and specific work-related aspects, and evaluating emotional states. These various approaches to measuring worker well-being highlight the complexity of the concept and the need for a comprehensive understanding. Additionally, by considering fluctuations over time and differentiating between broader life experiences and specific work-related aspects, researchers can capture the dynamic nature of worker well-being and its multidimensional nature. The review highlights the widespread use of transformative leadership. Additionally, the paper delineates an examination of many leadership styles, including authentic, ethical, and servant leadership, and the exploration of emerging or less investigated leadership styles such as respectful, inclusive, humble, virtuous, and responsible leadership.

Regarding research question 3, the field is heavily guided by four major theoretical frameworks including conservation of resource theory [115], self-determination theory [36], job demand and resource model [116] and Broaden and Built theory [117] to explain how positive leadership impacts workers’ well-being. These theories underscore leadership behaviours, resources, intrinsic motivation, and positive emotions as mechanisms through which favourable work environments are created to support improving workers' overall happiness and well-being. They highlight the importance of leaders creating a supportive and empowering work environment, providing necessary resources for employees, fostering intrinsic motivation, and promoting positive emotions among workers.

In research question 4, the findings show various types of positive leadership were consistently linked to positive impacts on different facets of employee well-being. However, the findings pertaining to authentic and transformational leadership exhibited a greater degree of variability, with conflicting results across a few studies. A considerable proportion of the research under evaluation primarily focused on investigating mediating factors, such as the meaningfulness of work, role ambiguity, and team environment, in the association between good leadership and the well-being of workers. Similarly, a few articles investigated moderating variables. Quite a high proportion of the mediating and moderating variables exhibited a statistically significant positive impact, suggesting their importance in comprehending the mechanisms through which positive leadership affects employee well-being through diverse pathways. Furthermore, these variables underscore their capacity to either strengthen or alter the association between positive leadership and worker well-being, particularly in specific contexts.

### 4.2 Suggestion for future research

The review of earlier studies has significantly improved the comprehension of the nexus between positive leadership and worker well-being. Given the growing popularity of positive leadership worldwide, the number of studies is anticipated to increase. Consequently, future research directions should aim at supporting rigorous theoretical and empirical research efforts to gain a better understanding of the relations between the two concepts.

First, the use of qualitative approach is considered a strength, as qualitative data collection and analysis methods provide a comprehensive understanding of research outcomes compared to quantitative studies [118]. It measures reality, focusing on the comprehension and explanation of dynamics of relations at the workplace [119]. The review suggests only 4 studies adopted qualitative approach. It is our contention that well-being is a pattern of behavioural processes and can best be explained using an in-depth approach. Consequently, future studies must endeavour to use more qualitative design, which would guarantee the gathering of real life and situations regarding positive leadership and wellbeing [120,121]. Additionally, the qualitative may help to explain the processes underlying specific causal relations regarding positive leadership and wellbeing [122].

Second, longitudinal designs permit researchers to assess the long-term effect of the relationship between independent and dependent variables [123] and are a relevant consideration for all quantitative and qualitative research designs [124]. Yet only 9 studies adopted the longitudinal approach. Additionally, all the nine studies were quantitative in nature. Due to emerging interest in positive leadership and worker well-being, comprehension of long-term impact is vital for organizations. Consequently, future research should consider both qualitative and quantitative longitudinal design. The use of longitudinal approach will help scholars to gain understanding of the long-term complex interplay effect of positive leadership on worker well-being [125]. Additionally, the few studies used relatively shorter time lags. This only provided a snapshot of well-being, given the career of individuals spanned over 40 years. Future studies must factor in longer time periods, in order to comprehend the development of well-being across longer time periods.

Third, sample adequacy in both quantitative and qualitative research relates to the appropriateness of the sample size and composition [126]. This is a significant consideration in the assessment of quality and trustworthiness of research outcomes [127]. The review suggests that quite a sizeable number of studies have employed small sample sizes. The use of small sample size in quantitative studies is a well-known threat to the statistical conclusion, validity and restricts generalizability [128]. Future studies should target a large sample size in the design stage of their studies. Additionally, to lower dropout rates, efforts must also be made to get a high response rate during the data collection phase.

Fourth, the adoption of mixed design to examine a phenomenon is advocated for as a means of gaining a better understanding of the contradictions between qualitative and quantitative data [129]. The use of a mixed approach is expected to offer a comprehensive, valid and dependable result, as evidence originates from different sources and findings are based on separate analytical procedures [130]. The review reveals that only 3 studies adopted a mixed method design. The use of mixed method research design is therefore encouraged in future studies. It is worthy to note that statistical survey methods cannot alone provide a comprehensive understanding of worker well-being in a positive leadership context. A mixed method approach provides participants the opportunities to have a strong voice and share their experiences throughout the study and opportunity to evaluate studies from a different perspective [131].

Fifth, the adoption of multilevel approach when studying worker well-being within positive leadership contexts is encouraged. Exercising of leadership within organization entails a dynamic interaction between followers, peers, customers, leaders, practitioners and situational context [132]. Future studies should investigate a multilevel view of individuals, dyadic relationships and team/organization using both qualitative and quantitative approaches.

Six, the review revealed, a few studies adopted several theories, including the conservation of resource theory, self-determination theory, job demand and resource model, and broaden and build theory to successfully establish a connection between positive leadership behaviours and employee perception of well-being. Majority of these theories examined leadership behaviours as a resource that supports the effective well-being of workers. We believe there is a greater need for theory engagement in the current positive leadership and worker well-being literature. This call is also relevant, since the field is at its nascent stage. For example, from the longitudinal design perspective, researchers can engage further theories such as set points theory [133] and dynamic equilibrium model [134] to explain changes in well-being over the cause of individuals work life. Additionally, the call for the use of moderating and mediating variables should enhance the adoption and replicating of theoretical frameworks from other fields of psychology.

Seventh, while the use of mediating variables has been significant, moderating variables have received less attention. Wright et al. [135], Wall and Wood [136], likewise Wu and Zambo [137], have advocated for the use of moderators and mediators to enhance our understanding of the causal relationship between variables. As a developing field, researchers in the future are encouraged to examine mediating and moderating variables within positive leadership and worker well-being.

### 4.3 Suggestion for practice

The insights derived from this review study also prompt us to provide practical suggestions. First, it is recommended that organizations enhance their collaborative efforts with managers, providing them with help in cultivating the requisite skills and behaviours associated with positive leadership. This can be achieved through a multifaceted approach that combines targeted training, personalized development, feedback mechanisms, and a conducive organizational culture [138,139].

For targeted training, it is imperative for organizations to offer structured programmes that are specifically tailored to cultivate the requisite competencies and knowledge essential for positive leadership behaviours [140]. The training programme should include a range of topics, including but not limited to communication, dispute resolution, decision-making, and team development. Additionally, the training programme should incorporate practical exercises and real-life case studies to allow leaders to apply their learning in a simulated workplace setting [141].

In the case of personalized development, organizations should provide tailored plans that are grounded in individual assessments, career objectives, and performance evaluations. Additionally, organizations should establish coaching programmes and individualized learning pathways to support each manager's growth [142]. These coaching programmes can help managers identify their strengths and areas for improvement, while the individualized learning pathways ensure that they receive the necessary training and resources to enhance their skills.

With feedback mechanisms, organizations should establish feedback loops that include performance evaluation, 360-degree assessments, periodic check-ins, or even anonymous surveys that allow inputs to be received from diverse perspectives, including peers, subordinates, and superiors. These feedback mechanisms are crucial for identifying areas for growth, addressing any concerns or issues, and promoting a culture of continuous improvement [143]. To foster a conducive organizational culture, it is important for organizations to promote attributes such as openness, trust, creativity, and cooperation.

Organizations should consider implementing mentorship programmes that pair managers with experienced leaders who can provide guidance and support in developing their leadership abilities [144,145]. This mentorship can help managers gain valuable insights, learn from real-world experiences, and navigate challenges more effectively. Additionally, creating opportunities for managers to network and exchange ideas with peers in similar roles can further enhance their leadership development journey.

To further positive leadership research, it is essential for academics to collaborate with industry professionals to collectively determine variables in need of investigation in positive leadership research. The involvement of organizations in providing their insights and requirements is crucial, as it enables researchers to address practical demands in their study and guide research endeavours on positive leadership and organizational practices towards enhanced effectiveness.

### 4.4 Limitations of the study

Although the present review provides an overview of positive leadership and worker well-being in an organizational context, it has certain shortcomings. The exclusion of the Web of Science database and the inclusion and exclusion criteria used in the data retrieval process might have led to the elimination of articles that were not published in journals indexed in Scopus. In addition, other documents such as conference papers, dissertations, and non-English language papers were excluded. Consequently, the comprehensiveness of the reviewed dataset is questionable. This notwithstanding, the Scopus and Google Scholar databases provide adequate coverage of essential literature.

## Funding statement

This study did not receive any specific grant from funding agencies in the public, commercial, or not-for-profit sectors.

## Data availability statement

Data will be made available on request.

## Additional information

No additional information is available for this paper.

## CRediT authorship contribution statement

**Edem M. Azila-Gbettor:** Writing – original draft, Methodology, Formal analysis, Data curation, Conceptualization. **Ben Q. Honyenuga:** Writing – review & editing, Methodology, Formal analysis, Conceptualization. **Eli A. Atatsi:** Writing – review & editing, Supervision, Project administration, Data curation, Conceptualization. **Christina Naa Ayertso Laryea:** Writing – review & editing, Project administration, Methodology, Data curation. **Abigail Nana Konadu Quarshie:** Writing – review & editing, Writing – original draft, Methodology, Data curation.

## Declaration of competing interest

The authors declare that they have no known competing financial interests or personal relationships that could have appeared to influence the work reported in this paper.
